# Structure-function analysis of TOPBP1’s role in ATR signaling using the DSB-mediated ATR activation in *Xenopus egg* extracts (DMAX) system

**DOI:** 10.1038/s41598-020-80626-1

**Published:** 2021-01-11

**Authors:** Katrina Montales, Ahhyun Kim, Kenna Ruis, W. Matthew Michael

**Affiliations:** 1 Molecular and Computational Biology Section, Department of Biological Sciences, University of Southern California, Los Angeles, CA 90089 USA; 2 Pharmacological Sciences PhD Program, University of California, Irvine, CA 92697 USA

**Keywords:** Biochemistry, Cell biology

## Abstract

The protein kinase ATR is activated at sites of DNA double-strand breaks where it plays important roles in promoting DNA end resection and regulating cell cycle progression. TOPBP1 is a multi BRCT repeat containing protein that activates ATR at DSBs. Here we have developed an experimental tool, the DMAX system, to study the biochemical mechanism for TOPBP1-mediated ATR signalling. DMAX combines simple, linear dsDNA molecules with *Xenopus* egg extracts and results in a physiologically relevant, DSB-induced activation of ATR. We find that DNAs of 5000 nucleotides, at femtomolar concentration, potently activate ATR in this system. By combining immunodepletion and add-back of TOPBP1 point mutants we use DMAX to determine which of TOPBP1’s nine BRCT domains are required for recruitment of TOPBP1 to DSBs and which domains are needed for ATR-mediated phosphorylation of CHK1. We find that BRCT1 and BRCT7 are important for recruitment and that BRCT5 functions downstream of recruitment to promote ATR-mediated phosphorylation of CHK1. We also show that BRCT7 plays a second role, independent of recruitment, in promoting ATR signalling. These findings supply a new research tool for, and new insights into, ATR biology.

## Introduction

**Figure 1:**
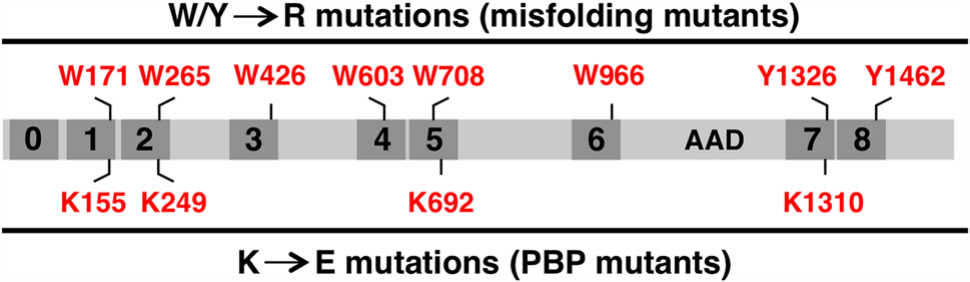
Schematic of TOPBP1 highlighting the BRCT domains and the AAD. We produced two panels of mutants. Misfolding mutants are shown up top and the PBP mutants are shown at the bottom.

When cells experience either DNA replication stress or DNA double-strand breaks (DSBs) they activate the ATR protein kinase^1,2^. ATR activation sets in motion numerous pathways that promote cell cycle delay, DNA repair, and processing of stalled replication forks. One direct substrate for ATR is the CHK1 protein kinase and phosphorylation of CHK1, on either serine 317 or 345 in the human protein, is often used as a marker for ATR activation^1,2^. Critical to ATR activation is the TOPBP1 protein—a large (~ 180 kDa) scaffold that contains nine copies of the BRCT domain (3, Fig. 1). BRCT domains are interaction modules that allow assembly of multi-protein complexes at sites of DNA damage^4–6^. One way BRCT domains can interact with other proteins is through a phosphate-binding pocket (PBP) that a subset of BRCT domains contains^5^. The PBP allows interaction with phosphorylated substrates by physically interacting with the phosphate moiety on the modified binding partner, and this allows the binding of BRCT domains to their partners to be regulated by protein kinases and phosphatases. It was not known which of TOPBP1’s many BRCT domains are required for ATR signaling, and one goal of this study was to close this knowledge gap.

Another functional region within TOPBP1 is its ATR Activation Domain (AAD; 7), which directly interacts with ATR, and the ATR binding partner ATRIP, to stimulate ATR kinase activity. The ATR pathway has been well studied over the years and some details of how it is activated have emerged^1,2^. The platform for ATR activation is RPA-coated single-stranded DNA (RPA-ssDNA) containing a 5′-DNA junction, a structure that is generated at both stalled forks and DSBs^1,2,8^. Critical factors that are recruited to this DNA structure include TOPBP1, ATR-ATRIP, and the 911 clamp protein^8–11^. RAD9 is one component of the 911 clamp, and it binds to TOPBP1’s BRCT1 domain at sites of DNA damage. The interaction between RAD9 and TOPBP1 is not required for ATR activation per se, but it is required for ATR to access a subset of its substrates, including CHK1^12^. Other factors also bind TOPBP1 in its BRCT1&2 region, and these include MDC1 and 53BP1^13,14^. The interaction with MDC1 bridges TOPBP1 to the MRN complex, as well as the ATM protein kinase, and this is thought to be important for ATR signaling at DSBs^13,15,16^. The interaction with 53BP1 also appears to be important for ATR signaling, but in a limited manner that is specific for the G1 phase of the cell cycle^14^. Thus while there is a fair amount known about factors binding to TOPBP1’s BRCT1&2 domains during a DSB response, there is comparatively little known about how the remaining 6 BRCT domains are involved, or if they are even needed for ATR signaling at DSBs.

A detailed mechanism for how ATR is activated in any context, stalled replication forks, ongoing replication, or DSBs, has yet to be delineated. One issue with this has been that several of the key players, including TOPBP1 and ATR, are essential for cell viability^1,2^. Thus when these factors are depleted from living cells, for loss of function studies, all subsequent analysis is done against the background of a sick and dying cell. Another issue is that the site of ATR activation in vivo, the chromosome, is a complex and poorly defined entity. In addition, TOPBP1 performs many functions that are independent of ATR signaling, for example initiating DNA replication, transcriptional control, and DNA repair at mitosis^3,17^, and thus pleiotropic effects of depleting TOPBP1 can render analysis difficult. To get around some of these issues we have, in this work, developed a new system to study ATR activation by TOPBP1. The system combines *Xenopus* egg extracts with simple and well-defined DNA templates that mimic DSBs. We go on to use this system to perform a structure–function analysis of TOPBP1’s function in ATR signaling.

## Results

### Characterization of the DMAX system

**Figure 2:**
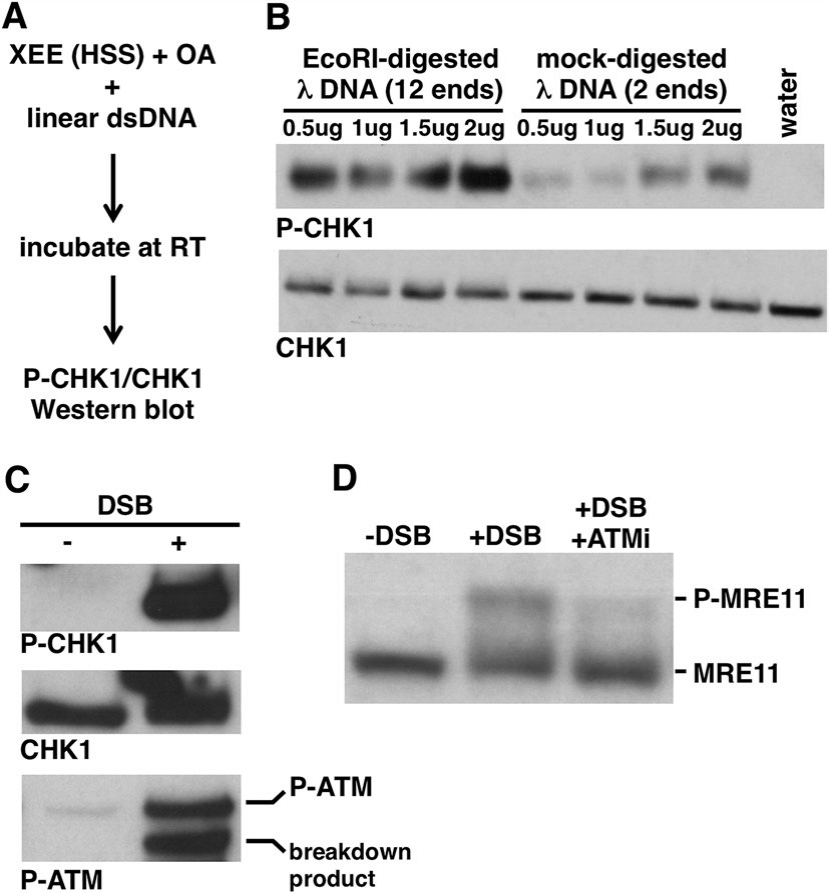
Linear dsDNAs trigger a legitimate DSB response upon incubation in HSS. (**A**) Experimental scheme. (**B**) The indicated amount of lambda DNA was incubated in 20 μl of HSS for 60 min. Samples were then probed by Western blot for P-CHK1 and CHK1. The sample labeled “water” did not receive any DNA. (**C**) 4 µg of EcoRI-digested lambda DNA (“DSB”) was optionally added to 20 μl of HSS and samples were processed as in (**A**). Samples were then probed for P-CHK1, CHK1, and P-ATM (P-Ser1981). (**D**) Either DMSO (lanes 1 and 2) or ATMi (KU55933, 50 µM, lane 3) was added to HSS, which was then optionally supplemented with EcoRI-digested lambda DNA (“DSB”). Samples were processed as in (**A**) and then probed for MRE11.

We sought a simple and well-defined experimental system to study the mechanism of TOPBP1-mediated control of ATR kinase during a DSB response. We settled on *Xenopus* egg extracts (XEEs) as the source of proteins and linear dsDNA as the source of DSBs (Fig. 2A). XEEs have a long and productive history in the analysis of ATR signaling^18,19^, and have been combined with a variety of DNA substrates, including demembranated sperm chromatin, AT70 (70-mer oligonucleotides of poly-A and poly-T annealed together), and circular M13 DNA with primers annealed to it (reviewed in 18). We chose linear dsDNAs as the source of DNA damage for our experiments because they are structurally similar to a broken chromosome, well defined, and easy and inexpensive to prepare. We used the high-speed supernatant (HSS) form of XEEs for these experiments. HSS is produced via ultracentrifugation of crude extract to fractionate the soluble proteins away from membrane vesicles. HSS has the advantage that it can be frozen and stored at − 80C and it retains activity upon thawing. Thus multiple experiments can be run with the same batch of extract, thereby reducing variability. In addition, HSS in conjunction with linear dsDNA templates has been used extensively in the past to study DNA end resection, which readily occurs in this system^20,21^, as well as for the study of ATM signaling^22,23^. Lastly, upon incubation in HSS, linear dsDNAs are rapidly assembled into chromatin^24^, allowing for signaling events to be studied in the natural context of chromatin. Thus, by adopting the HSS/dsDNA system for the study of ATR signaling, we can develop a new tool for ATR biology, one with a solid foundation made of previous work on chromatin assembly, end resection, and ATM signaling.

To assess ATR activity we monitored phosphorylation of its critical substrate, CHK1, using an antibody that recognizes phospho-serine 344, a known ATR site within *Xenopus* CHK1^25^. Previous work using AT70 to activate ATR in XEE has shown that it is necessary to include inhibitors of protein phosphatase 2A (PP2A) in the assay in order to stabilize, and thus visualize, P-CHK1^26^. The reason PP2A inhibitors are required is that small DNAs do not support nuclear assembly in XEE. Thus phosphatases that are normally found in the cytoplasm, and partitioned away from P-CHK1 in the context of nuclear assembly, are exposed to P-CHK1 in the absence of nuclear assembly. For this reason we included the PP2A inhibitor okadaic acid (OA) at 1 µM in our assay system.

As detailed above, ATR is activated by a variety of structurally diverse DNA substrates in XEE. As our focus is on DSB-mediated signaling, we wanted to be certain that the ATR activation that occurs upon addition of linear dsDNAs to XEE reflects genuine DSB-activated signaling. One hallmark of DSB signaling is that the free DNA end is critical for the activation of signaling^27^, and thus we examined the requirement for DNA ends in our system. To do so we used phage lambda DNA as the source of dsDNA, and we performed either a mock digestion, to produce a substrate with 2 DNA ends per input DNA molecule, or an EcoRI digestion, to produce a substrate with 12 ends per input DNA molecule (there are 5 EcoRI sites within lambda DNA). Equivalent amounts of DNA were titrated into XEE and, after incubation, ATR signaling was assessed by probing for P-CHK1. As a loading control we also probed for unmodified CHK1, to ensure that equivalent amounts of sample were examined across a given experiment. As shown in Fig. 2B, mock-digested lambda DNA activated ATR in a dose-dependent manner, as inferred by the appearance of P-CHK1 in samples receiving DNA, relative to the sample that did not. Importantly, ATR was more efficiently activated by the EcoRI-digested sample (Fig. 2B), showing that DNA ends are important for signaling in this system.

Another hallmark of genuine DSB-mediated signaling is the activation of ATM, and indeed the HSS/dsDNA system has been used extensively to study ATM^18,22,23^. To be sure that ATM activation was happening under our conditions we optionally added EcoRI-digested lambda DNA to XEE and, after incubation, we probed the samples for the Ser1981-phosphorylated form of ATM (P-ATM). Previous work has shown that ATM autophosphorylates on Ser1981 when it is activated by DSBs^28^. We observed that addition of dsDNA to HSS activated ATM, as expected (Fig. 2C). We also examined another known DSB-mediated signaling event, phosphorylation of the MRE11 protein. Previous work has shown that the DSB-activated phosphorylation of MRE11 in XEE causes reduced migration of the protein after SDS-PAGE^29^, and this is what we observed (Fig. 2D, compare lane 1 to lane 2). MRE11 is phosphorylated by ATM in response to DSBs^30^, and we observed that the ATM inhibitor KU55933 (ATMi) efficiently reduced the amount of phosphorylated MRE11 produced in the reaction (Fig. 2D). Our data show that addition of linear dsDNA molecules to HSS triggers ATR activation (Fig. 2B). In addition, they demonstrate that this is genuine, DSB-mediated activation of ATR, as shown by the effect of increased DNA ends on ATR signaling (Fig. 2B), and the co-activation of the DSB-dependent kinase ATM (Fig. 2C,D). To our knowledge, this is the first demonstration that the HSS/dsDNA system is compatible with ATR activation, and as such we have nicknamed this system DMAX, for DSB-mediated ATR activation in XEE.

**Figure 3:**
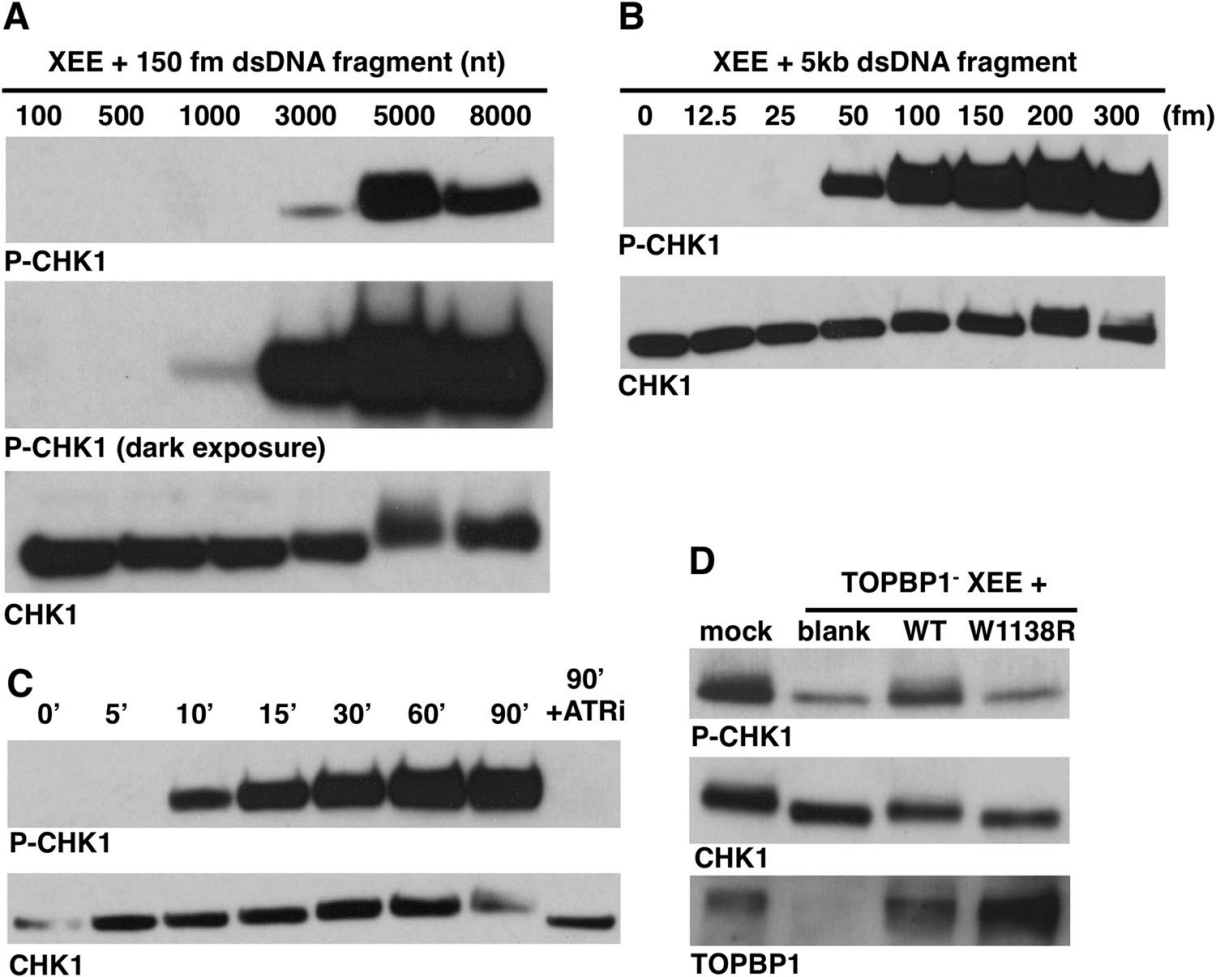
Basic properties of the DMAX system. (**A**) DMAX assay where dsDNA fragments of the indicated size (in nucleotides) were added to HSS at the indicated concentration. After 60-min incubation the samples were probed for P-CHK1 and CHK1. (**B**) DMAX assay where dsDNA fragments of the indicated sizeconcentration were added to HSS at the indicated concentration. After 60-min incubation the samples were probed for P-CHK1 and CHK1. (**C**) DMAX assay where HSS and 150 fmol of a 5kB dsDNA fragment were incubated together and samples were taken at the indicated time points. ATRi was included in the final sample, at 100 μM. Samples analyzed as in (**A**). (**D**) HSS was immunodepleted using either non-specific IgG (sample “mock”) or HU142, and antibody that recognizes TOPBP1 (samples “TOPBP1^-^ HSS + ”). The depleted extracts were then supplemented with either an unprogrammed IVTT reaction (sample “blank”) or IVTT reactions programmed for production of wild type TOPBP1 (“WT”) or the indicated TOPBP1 point mutant (“W1138R”). The reconstituted extracts were then supplemented with 150 fm of the 5 kB dsDNA fragment and, after incubation, the samples were probed by Western blotting for the indicated proteins. HU142, raised against *Xenopus* TOPBP1^44^, was used to probe for TOPBP1.

It was of interest to delineate some basic properties of DMAX. We first examined the length requirements for our dsDNA templates in ATR activation. For this we produced PCR fragments of varying sizes and confirmed that they were homogeneous in nature and of the correct size by DNA gel electrophoresis (data not shown). The different fragments were added to XEE, at equimolar amounts (150 fm), and after incubation ATR signaling was assessed by examining P-CHK1. As shown in Fig. 3A, maximal signaling occurred with fragments of 5 kb or longer. A darker exposure of the blot reveled that efficient signaling occurred with the 3 kb fragment, and that there was a steep drop-off to the next size down, 1 kb. P-CHK1 was not detected with the two smallest fragments (100 nt and 500 nt). This shows that maximal ATR signaling in DMAX requires at least 5 kb of input dsDNA. We next asked what the threshold concentration of 5 kb dsDNA would be for efficient ATR activation. As shown in Fig. 3B, 50 fm could activate signaling, and signaling was increased from there until reaching a maximal level at 150 fm. Moving on, we next examined the kinetics of ATR activation in the system by running a time-course experiment where the input DNA was 150 fm of the 5 kb PCR fragment (Fig. 3C). P-CHK1 was first detected at 10 min, and maximal signal was observed by 60 min. To be sure that the P-CHK1 we are monitoring is due to phosphorylation of CHK1 by ATR, an ATR inhibitor (ATR-45, ref. 31) was included, and this prevented the appearance of P-CHK1 (Fig. 3C, lane 8). These data show that 150 fm of a 5 kb dsDNA fragment and a 60-min incubation period are three optimal parameters for DMAX, and for the remainder of this study we exclusively use the 5 kb fragment as our source of DSBs, and all incubations are of one hour in duration.

In a final control experiment we wanted to be certain that TOPBP1 is required for DMAX. For this we performed immunodepletion, where either non-specific or anti-TOPBP1 antibodies are coupled to Protein A beads and then incubated with XEE. After incubation the XEE is recovered away from the antibody beads containing the captured TOPBP1 and used for DMAX. We also prepared recombinant forms of TOPBP1, using in vitro transcription and translation (IVTT) in rabbit reticulocyte lysates, so that they could be added back to the depleted extracts. As shown in Fig. 3D, a mock-depleted extract (treated with non-specific antibody beads, lane 1) contained TOPBP1 and was able to activate ATR upon addition of dsDNA. By contrast, for the TOPBP1-depleted extract, TOPBP1 levels were substantially reduced and this extract was deficient for ATR activation (Fig. 3D, lane 2). When wild type TOPBP1 was added back to the TOPBP1-depleted extract, then ATR activation was restored (lane 3), and this did not occur when a mutant TOPBP1, containing a W to R mutation at position 1138 in the AAD, was added back (lane 4). Previous work has shown that the W1138R mutant cannot activate ATR^7^. This experiment shows that our DMAX system is compatible with depletion/add-back experiments, and thereby sets the stage for the detailed structure–function analysis of TOPBP1’s role in ATR signaling that is described below.

### Delineation of TOPBP1 BRCT domain requirements for recruitment to DSBs

**Figure 4:**
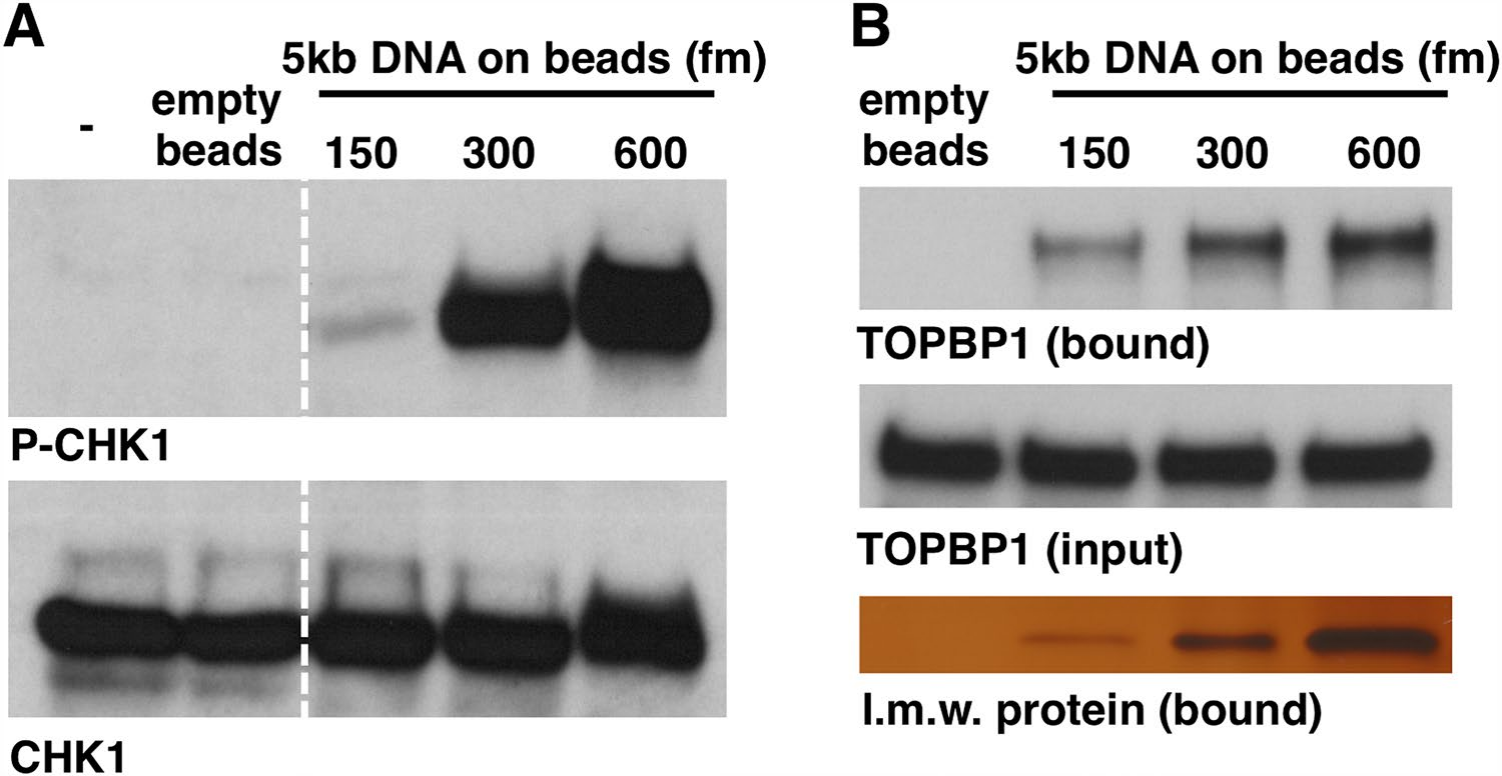
A DMAX-based DSB binding assay. (**A**) dsDNA (5kB) was coupled to magnetic streptavidin beads and then added to HSS at the indicated concentration of DNA. The sample labeled “–” received no beads. The sample labeled “empty beads” received only magnetic streptavidin beads. After a 60-min incubation samples were taken and probed for P-CHK1 and total CHK1. The dashed white line on the blots demarcates a lane from the original image that was removed because it is irrelevant. All samples shown were run on the same gel and blotted together on the same membrane at the same time. See Fig. S1 for the original scan. (**B**) dsDNA (5kB) was coupled to magnetic streptavidin beads and then added to HSS at the indicated concentration of DNA. One sample lacked DNA (“empty beads”). After a 60-min incubation the beads were isolated on a magnetic stand and the beads were then washed three times with PBS + 0.1% Triton X-100 and bound proteins were eluted using 2X Sample Buffer. Samples of the bound material, as well as the starting extracts (“input”) were then probed by Western blotting for TOPBP1. In addition, the bound samples were analyzed by silver staining, with a focus on a ~ 12.5 kDa low molecular weight (“l.m.w.”) showing that it is bound to the beads in a dose-dependent manner, as is TOPBP1.

One of the fundamental questions in ATR signaling is the mechanism by which its critical activator TOPBP1 is recruited to sites of damage. In principle, one could use DMAX to address this issue, by tethering the DSBs to magnetic beads as a way to isolate them back out of the extract to probe for TOPBP1 occupancy. To test this approach we first asked if attachment of a magnetic bead to one end of the linear dsDNA would allow ATR signaling. PCR fragments were generated containing a biotin moiety on end and the DNAs were then coupled to magnetic streptavidin beads to produce “DSB beads”. The DSB beads were added to XEE and, following incubation, the presence of P-CHK1 was assessed by Western blotting. As shown in Fig. 4A, the DSB beads were able to activate ATR, in a dose-dependent manner. We next asked if the TOPBP1 present in the XEE could stably associate with the DSB beads. Varying amounts of DSB beads were added to XEE and, after incubation, the beads were isolated, washed, and bound proteins were eluted. As shown in Fig. 4B, TOPBP1 could bind to the DSB beads, but not to empty beads. Thus, DSB beads can both recruit TOPBP1 and activate ATR. We also stained the DSB-bound material for total protein and observed that a low molecular weight protein of ~ 12.5 kDa, likely a histone, also associated with the DSB beads in a dose-dependent manner. Moving forward, we will use this protein as a control for equal isolation of the DSB beads between different samples within a given experiment.

**Figure 5:**
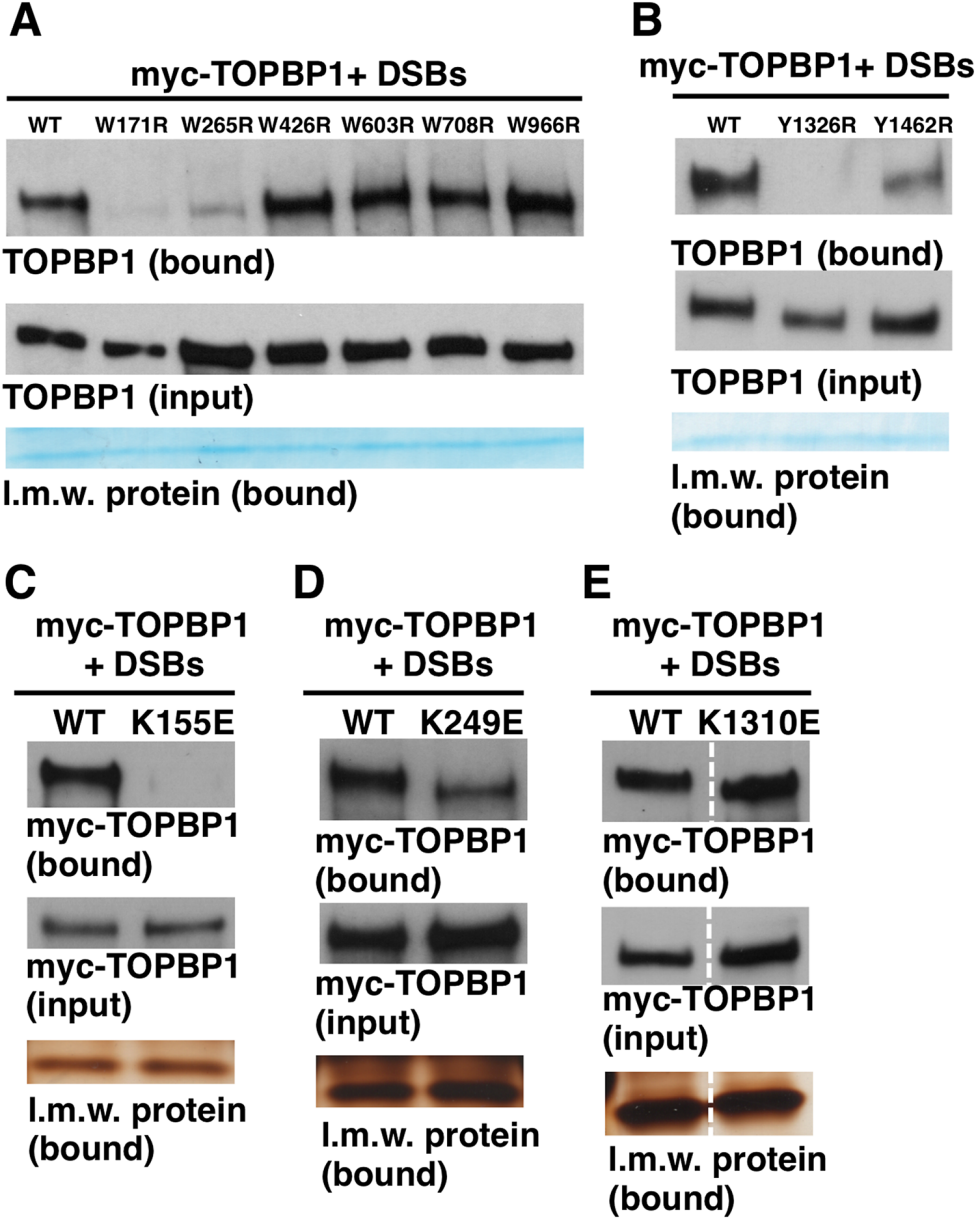
Mutational analysis of the roles of individual BRCT domains in recruitment of TOPBP1 to DSBs. (**A**) HSS was combined with IVTT-produced and myc-tagged TOPBP1 proteins. These proteins were the wild type form (“WT”) and the W to R BRCT domain misfolding mutants shown in Fig. 1. The numbers above the blot reflect the given BRCT domain containing the W to R mutation. DSB beads were then added and the samples were incubated for 60 min. After incubation, the beads were processed as in Fig. 3B and the samples were probed for the indicated protein. The myc antibody was used to probe for TOPBP1. The bound samples were also stained by Coomassie and the ~ 12.5 kDa l.m.w protein is shown. (**B**) Same as (**A**) except the Y to R BRCT misfolding mutants were analyzed. (**C–E**) Same as (**A**) except the indicated PBP mutant was analyzed. The l.m.w. protein was visualized by silver staining. For (**E**), the dashed white line on the blots demarcates a lane from the original image that was removed because it is irrelevant. All samples shown were run on the same gel and blotted together on the same membrane at the same time. See Fig. S1 for the original scan.

The major goal of this study was to identify which of TOPBP1’s many BRCT domains are important for its role in activating ATR at DSBs. While previous work has approached structure–function analysis of TOPBP1 by simply deleting individual BRCT domains within the protein^32^, we took a different approach. We utilized a previously described panel of TOPBP1 BRCT domain “misfolding” mutants (Fig. 1), whereby individual BRCT domains were inactivated by mutation of a highly conserved hydrophobic residue within the given domain^33^. These residues, tryptophans in BRCTs 1–7 and tyrosines in BRCTs 7&8 (Fig. 1), are buried within the hydrophobic core of the BRCT domain^34^, and mutation to a charged residue such as arginine is likely to cause deleterious misfolding and inactivation of the domain. We note that BRCT0 lacks this hydrophobic residue. Having established a DSB binding assay we next subjected our panel of misfolding mutants to the assay in order to determine which BRCT domains are needed for DSB association. For this, myc-tagged TOPBP1 proteins were produced by IVTT and then added to XEE, along with DSB beads. After incubation the beads were isolated, washed, and probed for TOPBP1 occupancy using the myc antibody. As shown in Fig. 5A, mutations in BRCT domains 1 and 2, but not mutations in domains 3–6, prevented efficient binding of TOPBP1 to DSBs. Interestingly, we also observed that a misfolding mutation in BRCT7 prevented efficient binding to DSB beads, while a similar mutation in BRCT8 reduced, but did not eliminate, binding (Fig. 5B). These data point to the BRCT0-2 and BRCT7&8 regions as important for TOPBP1 recruitment to DSBs.

Data obtained from the misfolding mutants identify BRCT domains 1,2, and 7 as important for TOPBP1 recruitment to DSBs. All of these BRCT domains contain a PBP, and it was thus of interest to determine if phosphate binding by these BRCT domains was needed for TOPBP1 recruitment. The PBP contains a conserved lysine residue that we mutated to an oppositely charged residue, glutamic acid (Fig. 1). As shown in Fig. 5C–E, just one PBP mutant failed to bind DSBs efficiently, the mutant corresponding to BRCT1. Based on these data, we conclude that TOPBP1 recruitment to DSBs involves a phosphorylation-dependent interaction between BRCT1 and a binding partner present at the DSB, as well as a phosphorylation-independent interaction involving BRCT7.

### Delineation of TOPBP1 BRCT domain requirements for ATR-mediated phosphorylation of CHK1

**Figure 6:**
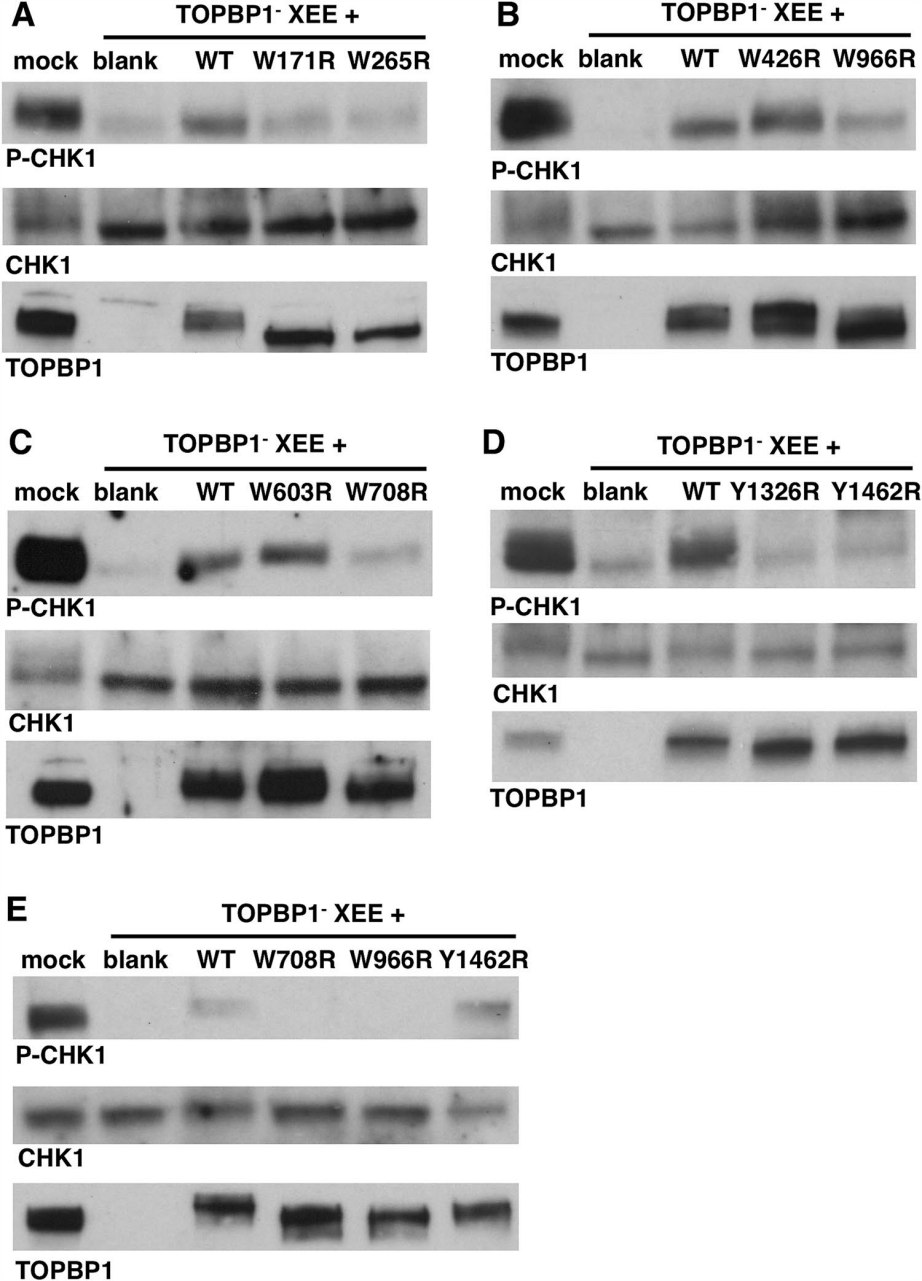
Multiple TOPBP1 BRCT domains are required for ATR-directed phosphorylation of CHK1. **A−E** DMAX assays where HSS was depleted of endogenous TOPBP1 and then supplemented with the indicated IVTT-produced TOPBP1 mutants prior to the addition of DSBs. Mock refers to extract that was mock-depleted using non-specific IgG. Blank refers to a TOPBP1-depleted extract that received an unprogrammed IVTT reaction. After incubation, the samples were probed by Western blotting for P-CHK1, CHK1, and TOPBP1 (using anti-TOPBP1 antibody HU142).

Having established the BRCT requirements for recruitment to DSBs we moved on to a downstream step, ATR-mediated phosphorylation of its critical substrate CHK1. For this we used immuno-depletion and add-back, as was done in Fig. 3D. We first examined the panel of misfolding mutants and observed that mutations in BRCT domains 1, 2, 5, and 7 all consistently prevented efficient phosphorylation of CHK1 (Fig. 6A–D). The misfolding mutants in BRCT6 (W966R) and BRCT8 (Y1426R) gave more variable results, where in some experiments the mutants could rescue CHK1 phosphorylation in TOPBP1-depleted extract (see Fig. 6B for W966R and Fig. 6E for Y1462R), whereas in other experiments rescue was not observed (Fig. 6E for W966R and Fig. 6D for Y1462R). We conclude that BRCT domains 1, 2, 5, and 7 are essential for ATR-mediated phosphorylation of CHK1 and that BRCT domains 6 and 8 make the process more efficient.

**Figure 7:**
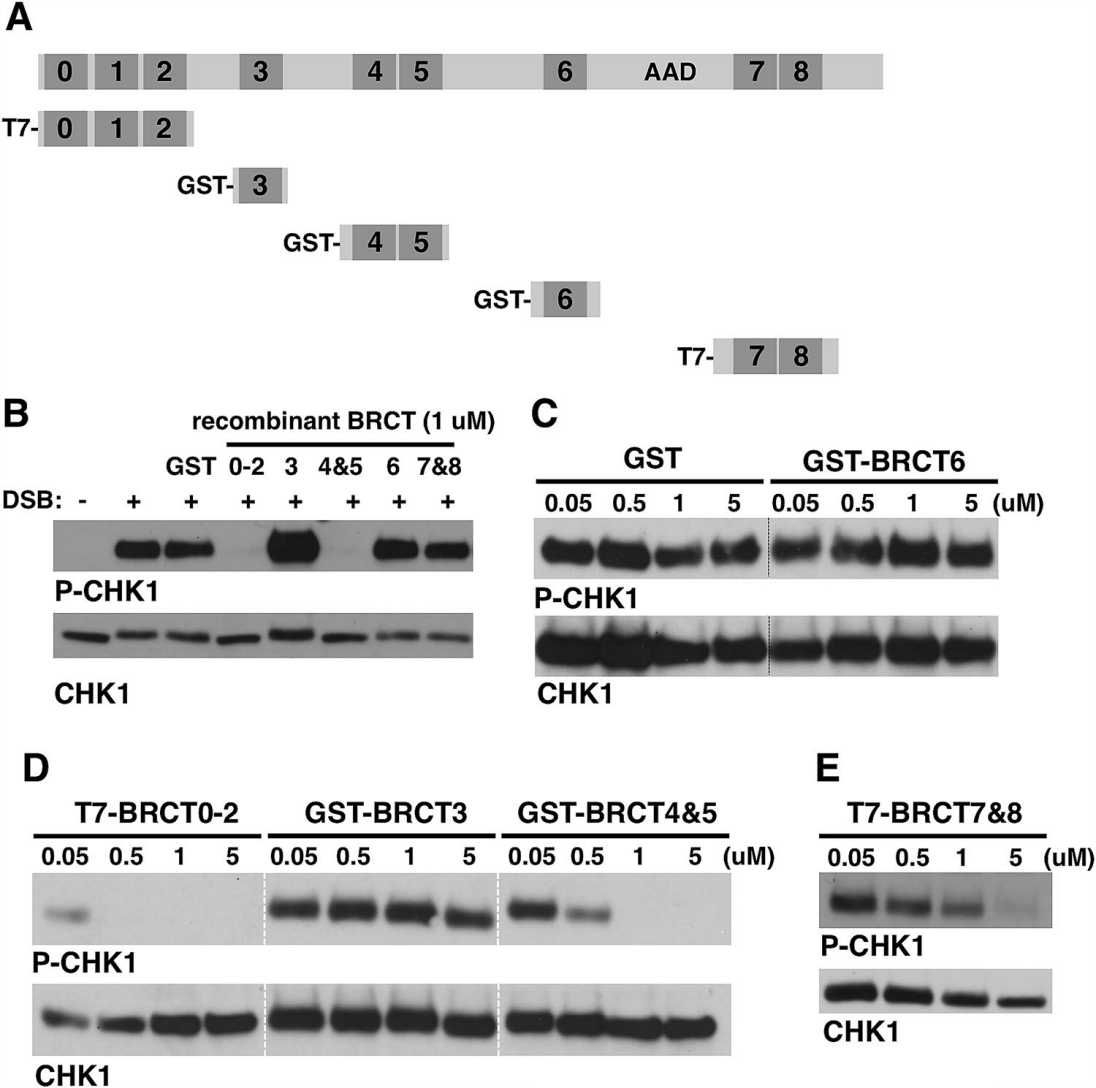
Addition of isolated BRCT domains in excess blocks ATR signaling. (**A**) Schematic representation of full-length TopBP1 and the recombinant GST-tagged TOPBP1 BRCT domains used in (**B**). (**B**) Overexpression assay where the indicated purified proteins were added to HSS at a 50-fold excess over endogenous TOPBP1 and preincubated for 15 min. DSBs were then added to the samples and incubated for 60 min. Samples were probed by Western blotting for P-CHK1 and CHK1. The sample in lane 1 did not receive DSBs. (**C–E**) Same as (**B**) except that indicated proteins were added to the assays at the indicated concentration. For (**D**), the dashed white line on the blots demarcates a lane from the original image that was removed because it is irrelevant. All samples shown were run on the same gel and blotted together on the same membrane at the same time. See Fig. S1 for the original scan.

As an independent test for TOPBP1 BRCT domain function during ATR signaling we next asked if addition of isolated BRCT domains, in excess, to the XEE would perturb the DMAX reaction. The rationale is that the isolated domains would bind their natural partners and thereby titrate them away from endogenous TOPBP1 at the DSB. We thus prepared recombinant proteins (Fig. 7A), purified from *E. coli*, and added them to DMAX reactions at a concentration of 1 μM, which is a ~ 25-fold excess over the endogenous TOPBP1^35^. As seen in Fig. 7B, 1 μM T7-BRCT0-2 and GST-BRCT4&5 could block ATR signaling in the DMAX reaction, whereas the other proteins we tested, GST alone, GST-BRCT3, GST-BRCT6, and T7-BRCT7&8, did not inhibit the reaction. We next performed a more detailed titration of the proteins, and observed that even a high concentration of GST, GST-BRCT3, and GST-BRCT6 did not inhibit signaling (Fig. 7C,D). By contrast, relatively low amounts of T7-BRCT0-2 (50 nM) could inhibit signaling, while a bit more GST-BRCT4&5 was needed to attenuate signaling (500 nM, Fig. 7D). When we titrated T7-BRCT7&8 we found that it took more protein to eliminate ATR signaling than was the case for BRCT0-2 or BRCT4&5, but nonetheless signaling was blocked (Fig. 7E). These data confirm the point mutant analysis and show that the critical BRCT domains for TOPBP1’s function in ATR signaling are 0–2, 4&5, and 7&8.

**Figure 8:**
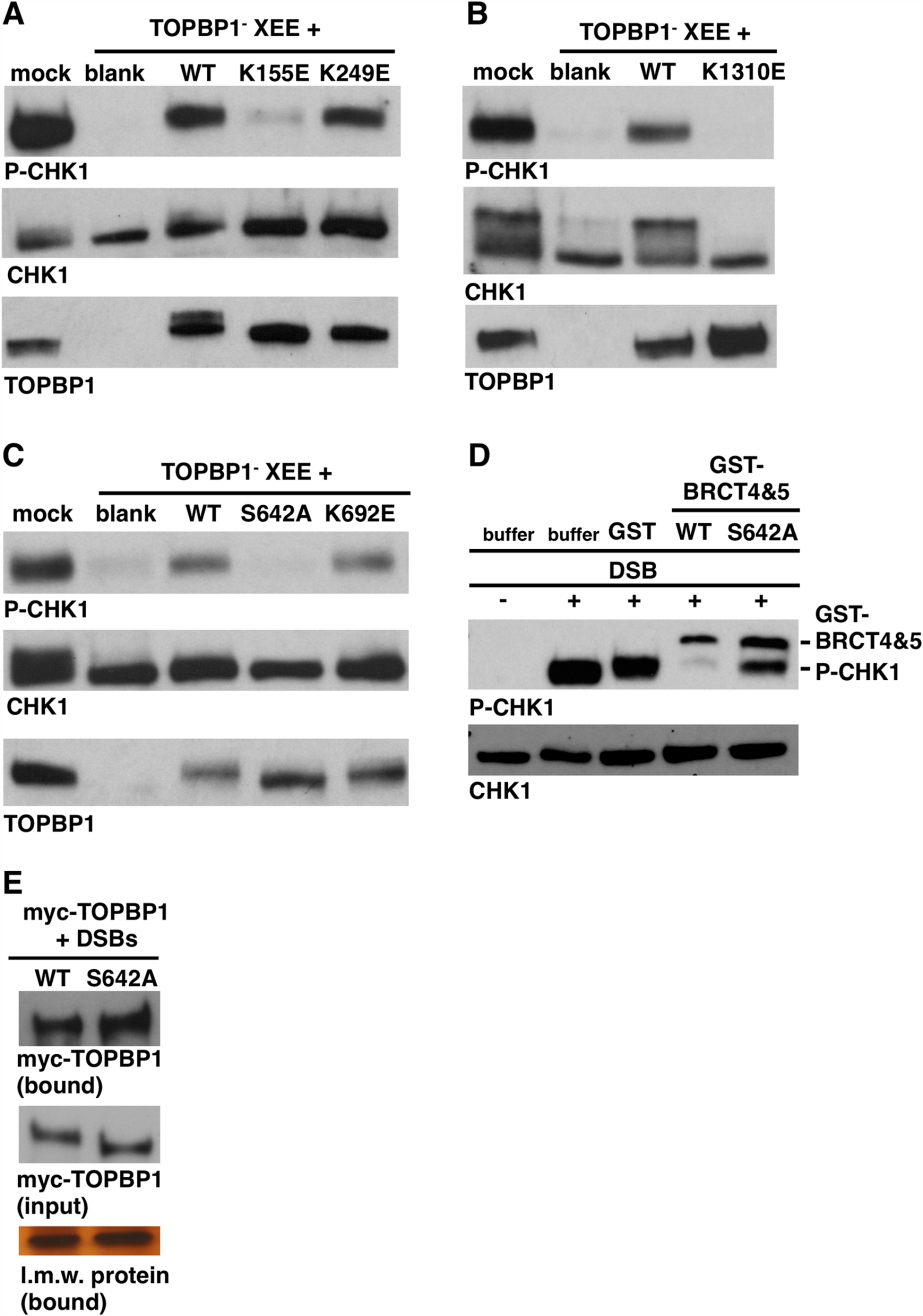
The role of TOPBP1’s PBPs in ATR signaling. **A–C** Immunodepletion and add-back DMAX assays were performed exactly as in Figs. 2E and 5A–D. The TOPBP1 PTP mutants were analyzed and are indicated above the blots. (**D**) A BRCT overexpression assay was performed using 1 μM of the indicated GST fusion protein. Samples were processed exactly as in Fig. 6. The GST-BRCT4&5 protein itself cross-reacts with some batches of the P-CHK1 antibody, and this band is labeled. (**E**) DSB binding assay with the indicated proteins. Samples were processed exactly as in Fig. 4.

All three of the BRCT domain sets that are required for ATR signaling contain PBPs, and thus we next asked which of the PBPs is needed for ATR-mediated phosphorylation of CHK1. As shown in Fig. 8A,B, PBP mutants in BRCT1 and BRCT7, but not BRCT2, abolished signaling. We next focused on the BRCT5 PBP. As shown in Fig. 8C, the K692E mutation did not prevent TOPBP1 from activating ATR. This residue corresponds to K704 in human TOPBP1, and has been shown to be required for phosphate binding by BRCT5^36,37^. Interestingly, mutation of S642, which is also part of the PBP^38^, totally prevented ATR activation (Fig. 8C). Indeed, when we performed a BRCT overexpression experiment, we found that while GST-BRCT4&5 in the wild type form could block ATR activation, a GST-BRCT4&5 protein harboring the S642A mutation could not (Fig. 8D). Based on this, it appears that while S642 is important for ATR signaling, the BRCT5 PBP itself is not. To see if S642 is required for TOPBP1 recruitment we tested the S642A mutant in our DSB binding assay and found that it could bind DSBs as well as the wild type protein (Fig. 8E).

## Discussion

In this work we present a new experimental tool for the study of DSB-mediated ATR signaling, the DMAX system. DMAX offers several advantages for studying ATR biology. One, DMAX is compatible with frozen XEE (HSS), and this allows many experiments to be performed with the same batch of extract, which is crucial for consistency in results. Two, the source DNAs for DMAX, commercially available lambda DNA or PCR fragments, are well defined, inexpensive, and easy to prepare. Three, DMAX is compatible with bead-bound DNA and its isolation back out of the extract, and this allows for studies on the recruitment of DDR factors to DSBs. Fourth, DMAX is compatible with immunodepletion and add-back experiments and, furthermore, proteins for add-back can be produced by IVTT. The ability to produce recombinant proteins by IVTT saves time, money, and labor and is more likely to result in active proteins than is expression and purification of recombinant proteins from bacteria or insect cells. Fifth, DMAX is compatible with small molecule inhibitors of DDR proteins, as shown here by our demonstration that the ATR inhibitor ATR-45 blocks DSB-mediated ATR signaling. Sixth, for loss of function experiments, proteins that are essential for cell survival, like TOPBP1 and ATR, can be removed from the XEE without concern about non-specific effects related to loss of viability. This is not true when these factors are depleted using siRNA in tissue culture cells. Thus, while people have used dsDNAs together with HSS in the past to study ATM, the novelty of the DMAX system presented here is its adaption to ATR signaling, its utility in allowing structure–function analysis of ATR regulators via immuno-depletion and add-back of IVTT-produced proteins, as well as its capacity for studying how ATR regulators are recruited to DSBs.

**Figure 9:**
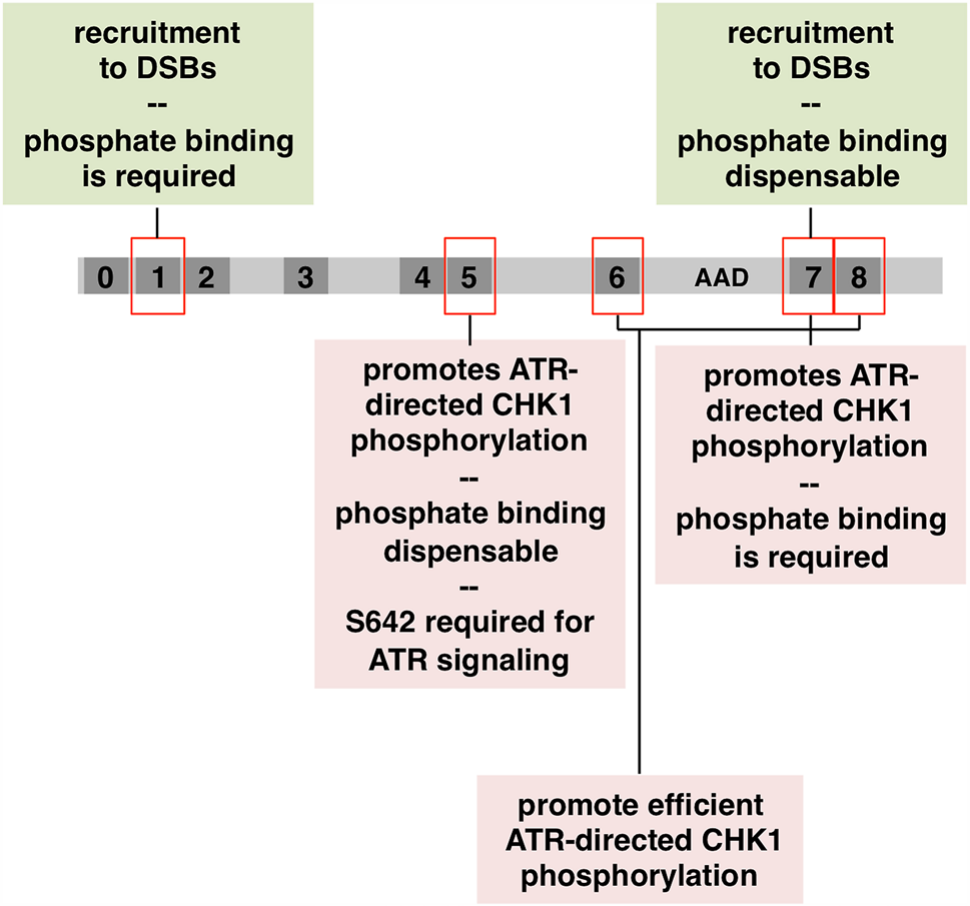
Summary of the TOPBP1 BRCT domains that participate in ATR signaling. Please see “Discussion” for details.

In this study we road-tested DMAX as a tool for structure–function analysis of the crucial ATR regulator TOPBP1. Using point mutants that disable distinct BRCT domains within TOPBP1 we found that inactivation of BRCT domains 1, 2, and 7 all prevent recruitment of TOPBP1 to DSBs (Fig. 5; summarized in Fig. 9). The BRCT1&2 region is a hotspot for TOPBP1 binding partners, and several factors that are known to accumulate at sites of damage can bind the TOPBP1 BRCT1&2 domains. These include RAD9, RHINO, MDC1, 53BP1, and topoisomerase II^3,17^. It is not currently known if any of these factors are required for TOPBP1 recruitment to DSBs. We have shown here that binding of BRCT1&2 to its target at DSBs is likely to require that the target be phosphorylated, as disruption of the BRCT1 PBP prevents recruitment. Interestingly, the BRCT2 PBP is not required for recruitment, however a BRCT2 misfolding mutant (W265R) does prevent recruitment. We feel it is likely that the W265R mutation impacts the integrity of BRCT1, as we have observed that the W265R mutant cannot bind to RAD9 (data not shown) and others have shown that BRCT1 represents the RAD9 binding site^11^. Thus it seems likely that the role of the BRCT1&2 region in recruitment of TOPBP1 to DSBs is played out via phosphorylation-dependent binding of BRCT1 to an as yet to be identified factor at the DSB. BRCT2 may also be involved, as our own previous work has shown that BRCT2 binds to RPA-ssDNA directly^39^. We also tested our BRCT1&2 mutants for the ability to promote ATR-mediated phosphorylation of CHK1, and all of the mutants that prevented TOPBP1 recruitment to DSBs (W171R, W265R, and K155E) also failed to support CHK1 phosphorylation, whereas the one mutant that allows DSB binding (K249E) is also permissive for CHK1 phosphorylation. This suggests that stable binding to DSBs is necessary for TOPBP1 to control ATR signaling, and that the phosphate binding capacity of BRCT2 is dispensable for TOPBP1’s role in ATR signaling at DSBs. Whether or not the BRCT1&2 region functions in events downstream of recruitment remains an open question.

Our analysis of the BRCT4&5 region also yielded important new insights. We observed that these domains are dispensable for recruitment to DSBs however they are required for downstream events leading to CHK1 phosphorylation (Fig. 9). Although BRCT5 contains a PBP, the pocket is not needed for BRCT5 to perform its function during ATR signaling, as inferred by our analysis of the K692E pocket mutant (Fig. 8C). Interestingly, however, S642, which is also part of the PBP, is required for ATR signaling (Fig. 8C,D). We note that S642 sits within an “SQ” motif, which is a site commonly phosphorylated by ATM and ATR kinases, raising the possibility that phosphorylation of S642 is important for signaling. To date the only known factors that bind TOPBP1’s BRCT4&5 region are the BLM helicase and 53BP1^14,36,37,40^. BLM plays an important role during end resection, and is thus present at DSBs. However, BLM is unlikely to be the relevant BRCT4&5 binding partner for ATR signaling as others have shown that the BRCT5 PBP is required for BLM binding^36,37^ and we have shown here that the BRCT5 PBP is dispensable for ATR signaling. It will thus be important for future work to identify the relevant BRCT4&5 binding partner for ATR signaling. Another interesting outcome of our analysis involves BRCT4, which we have found to be irrelevant for ATR signaling based on the W608R misfolding mutant (Figs. 5A, 6C). It is noteworthy that the W608R misfolding mutation does not impact the function of the neighboring BRCT5 domain, as might be expected given that the W265R BRCT2 mutant clearly impacts BRCT1 function, as detailed above. It thus appears that while the BRCT1&2 region is sensitive to misfolding mutants in nearby domains, the BRCT4&5 region is not.

Lastly, we have found that TOPBP1 BRCT7 plays at least two distinct roles in ATR signaling (Fig. 9). When the domain is ablated via the Y1326R misfolding mutant then both TOPBP1 recruitment to DSBs and CHK1 phosphorylation are compromised. Importantly, however, we find that the BRCT7 PBP mutant K1310E is proficient for DSB binding but not CHK1 phosphorylation. Thus, this separation of function mutant allows us to conclude that BRCT7 facilitates recruitment via a distinct mechanism than what is happening to promote CHK1 phosphorylation. Which proteins might interact with the BRCT7&8 region to promote ATR signaling? One obvious candidate is FANCJ/BACH1, which has been shown to interact with TOPBP1 at stalled replication forks and is also an important factor for end resection during DSB repair^32,41^. Another candidate is the WDR18 protein, which has previously been shown to bind BRCT7&8 and to play a role in ATR-mediated phosphorylation of CHK1^42^. In these studies it was shown that depletion of WDR18 abrogated, but did not eliminate, AT70-mediated ATR signaling. Thus it may be that WDR18, a WD-40 repeat protein, acts redundantly with another WD-40 family member to promote ATR signaling via TOPBP1’s BRCT7&8 domains.

In closing we’d like to address the possibility that multiple factors are likely to be interacting with any given TOPBP1 BRCT domain, at the same time, during ATR signaling. Indeed, two factors, RHINO and RAD9, are both involved in ATR signaling and they both bind the same site on TOPBP1, BRCT1’s PBP^43^. How can it be that two factors bind the same site at the same time during signaling? Recent work from our laboratory has solved this problem by showing that TOPBP1 functions as an oligomer^33^. Indeed, we have found that a given TOPBP1 oligomer can simultaneously bind both RAD9 and RHINO. This work also shows that TOPBP1’s AAD is active as a tetramer, and thus it is likely that the oligomeric form of TOPBP1 present at DSBs is a tetramer. If so, then the number of different factors that congregate on the TOPBP1 scaffold during ATR signaling could be substantial.

## Materials and methods

### Materials

#### Plasmids

*E. coli* expression vectors (all contain *Xenopus* TOPBP1 sequences):

GST-BRCT3 (pAK2, parental vector pGEX-4T3, aa coordinates 334–479).

GST-BRCT4&5 (pHG49, parental vector pGEX-4T3, aa coordinates 480–758).

GST-BRCT6 (pAK16, parental vector pGEX-4T3, aa coordinates 894–970).

GST-BRCT7&8 (pHG8, parental vector pGEX-4T3, aa coordinates 1197–1513).

T7-BRCT0-2 (pHS12, parental vector pET28A, aa coordinates 1–333).

T7-BRCT7&8 (pKR9, parental vector pET28A, aa coordinates 1247–1480).

IVTT expression vectors (all used pCS2 + MT as parental vector and all contained full-length *Xenopus* TOPBP1):

Wild type pCut5, K155E pAK85, W171R pAK22, K249E pAK60, W265R pAK27, W426R pHS2, W603R pHG26, S642A QC11, K692E pHG137, W708R pHG27, W966R pHS17, K1310E pAK99, Y1326R pHG55, Y1426R pHG51.

### Recombinant proteins

The recombinant proteins used in this study were GST, GST-BRCT 3, GST-BRCT 4&5, GST-BRCT 6, T7-HIS-BRCT0-2, and T7-HIS-BRCT7&8. All proteins were expressed in *E. coli* BL21(DE3) cells at 37 °C for 4 h and purified from the soluble fraction according to standard procedures. Details can be provided upon request.

### Antibodies

We used the following commercially sourced antibodies in this work: Myc (Millipore Sigma #M4439), GST (Millipore Sigma #05-782), CHK1 (Santa Cruz Biotechnology #sc-8408), P-CHK1 (Cell Signaling Technology #2341S), and P-ATM (ATM phospho S1981 Antibody Rockland # 200-301-400S). We also used our own antibody against *Xenopus* TOPBP1, HU142, which has been described^44^. The antibody against MRE11 was a kind gift of Howard Lindsay^45^.

### DNA substrates for DMAX assays

The major DNA substrate used for DMAX assays was a 5 kb PCR fragment that was produced in the following manner: 8.6 ng of template DNA (the pCut5 plasmid, volume 2 µl) was combined with 5 μl of 0.5 μM forward (5′- GCGAGTTACATGATCCCCC-3′) and 5 µl of 5 µM reverse (5′-AGCAATAGCATCACAAATTTCACAAATAAAGCATTTTTTTC-3′) primers along with 0.4 mM dNTPs (volume 4 µl), 20 µl of Phusion GC Buffer (NEB #B0519S),1 µL Phusion High-Fidelity DNA Polymerase (NEB #M0530S), and 63 µl of water. The annealing temperature was 54.5 °C with a 5-min extension time. For DSB binding assays the forward primer contained a biotin group on the 5′ end. We note that Phusion High-Fidelity DNA Polymerase produces blunt-ended fragments. After PCR the DNAs were purified using a Qiagen QIAquick PCR Purification Kit, according to the manufacturer’s instructions. The sequence of the 5 kb PCR fragment is available upon request. For Fig. 3A, the remaining PCR fragments were made exactly as described above for the 5 kb fragment. Primer sequences for these additional fragments are available upon request. Lambda DNA, used in Fig. 2, was purchased from New England Biolabs (NEB# N3011S). After either mock or EcoRI digestion of the lambda DNA it was purified using a Qiagen QIAquick PCR Purification Kit, according to the manufacturer’s instructions.

### Methods

#### Xenopus egg extracts

HSS was prepared exactly as described^46^. Immunodepletion of TOPBP1 was performed as described^44^.

#### IVTT production of proteins

IVTT reactions were performed using the SP6 TnT Quick Coupled Transcription/Translation System (Promega #L2080) according to the manufacturer’s instructions. Proteins were not purified after their production by IVTT, rather, the entire IVTT reaction was used as the source of a given protein.

#### DSB-mediated ATR activation in XEE (DMAX) assay

For DMAX assays, okadaic acid (OA) was first mixed with 20 µL of HSS to a final concentration of 1 µM. Linear dsDNA was then added to the mixture and reactions were incubated at room temperature for 60 min. Samples were analyzed via Western blotting using standard conditions. For DMAX reactions containing IVTT-produced proteins we typically added 2.5 μl of the IVTT reaction to each DMAX reaction.

#### DSB-binding assay

The 5 kb PCR fragment was biotinylated via inclusion of a biotin moiety on the forward PCR primer. After PCR and cleanup, the 5 kb PCR fragment was coupled to magnetic streptavidin beads (Dynabeads M-270 Streptavidin, ThermoFisher) according to the manufacturer’s instructions. These “DSB beads”, containing 600 fmol of dsDNA per assay, were then incubated in 20 µl of HSS supplemented with 5 µl of an IVTT reaction programmed to produce the protein of interest. After incubation the beads were collected on a magnetic stand and washed three times in PBS + 0.1% TritionX-100. Bound proteins were then eluted with 2X SDS-PAGE sample buffer and examined by Western blotting.

## Supplementary information

Supplementary Information.
