# Supplementary material for: Structure-function analysis of TOPBP1’s role in ATR signaling using the DSB-mediated ATR activation in Xenopus egg extracts (DMAX) system
Source: Sci Rep. 2021 Jan 11;11:467. doi: 10.1038/s41598-020-80626-1 (PMC7801695; doi:10.1038/s41598-020-80626-1)
Supplement: Supplementary file 1 — Supplementary Information. [file 41598_2020_80626_MOESM1_ESM.docx]

**Supplemental Information**

**Structure-function analysis of TOPBP1’s role in ATR signaling using the DSB-mediated ATR activation in *Xenopus* egg extracts (DMAX) system**

Katrina Montales^1^, Ahhyun Kim^1^, Kenna Ruis^1^, and W. Matthew Michael^1*^

Molecular and Computational Biology Section, Department of Biological Sciences, University of Southern California, Los Angeles, CA, 90089, USA

**Supplementary Figure Legends**

**Figure S1.** There were three instances where irrelevant material was removed from the gel scans (Figs 4A, 5E, and 7D). Shown here are the original images prior to removal of the irrelevant portions.

**Figure S2.** Raw scans for the blots shown in this work.


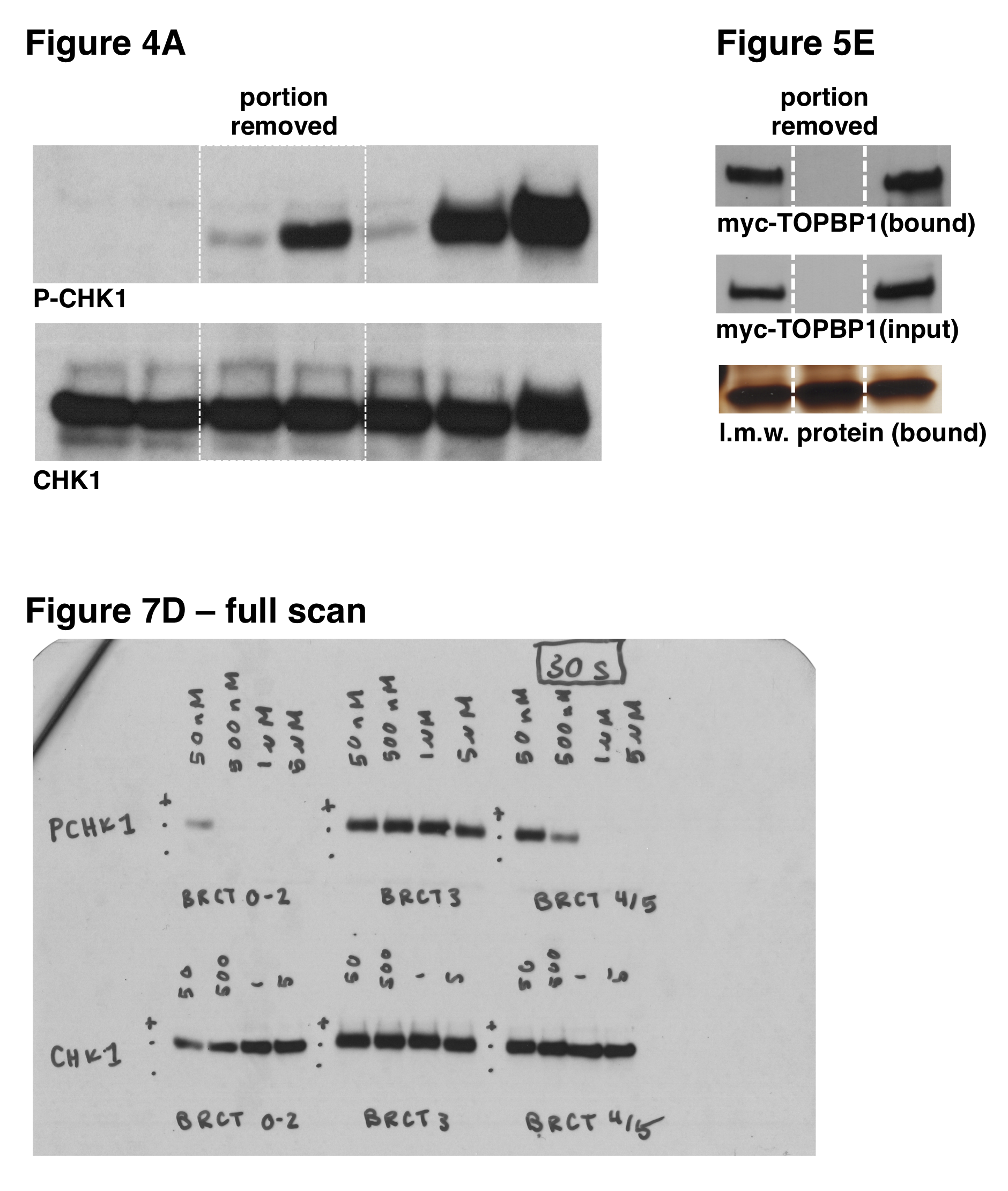


**Figure S1.**

**
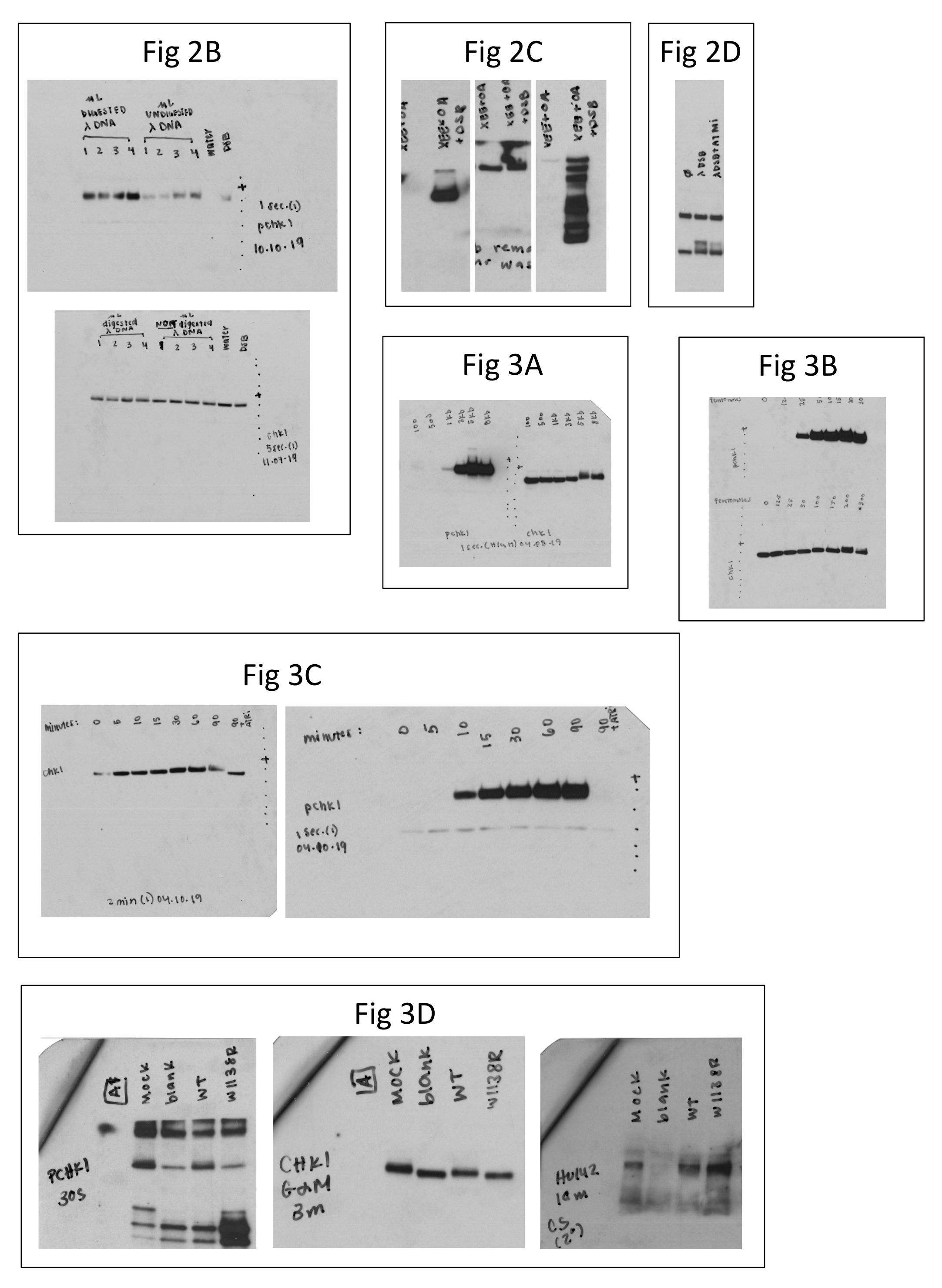
**

**Figure S2**

**
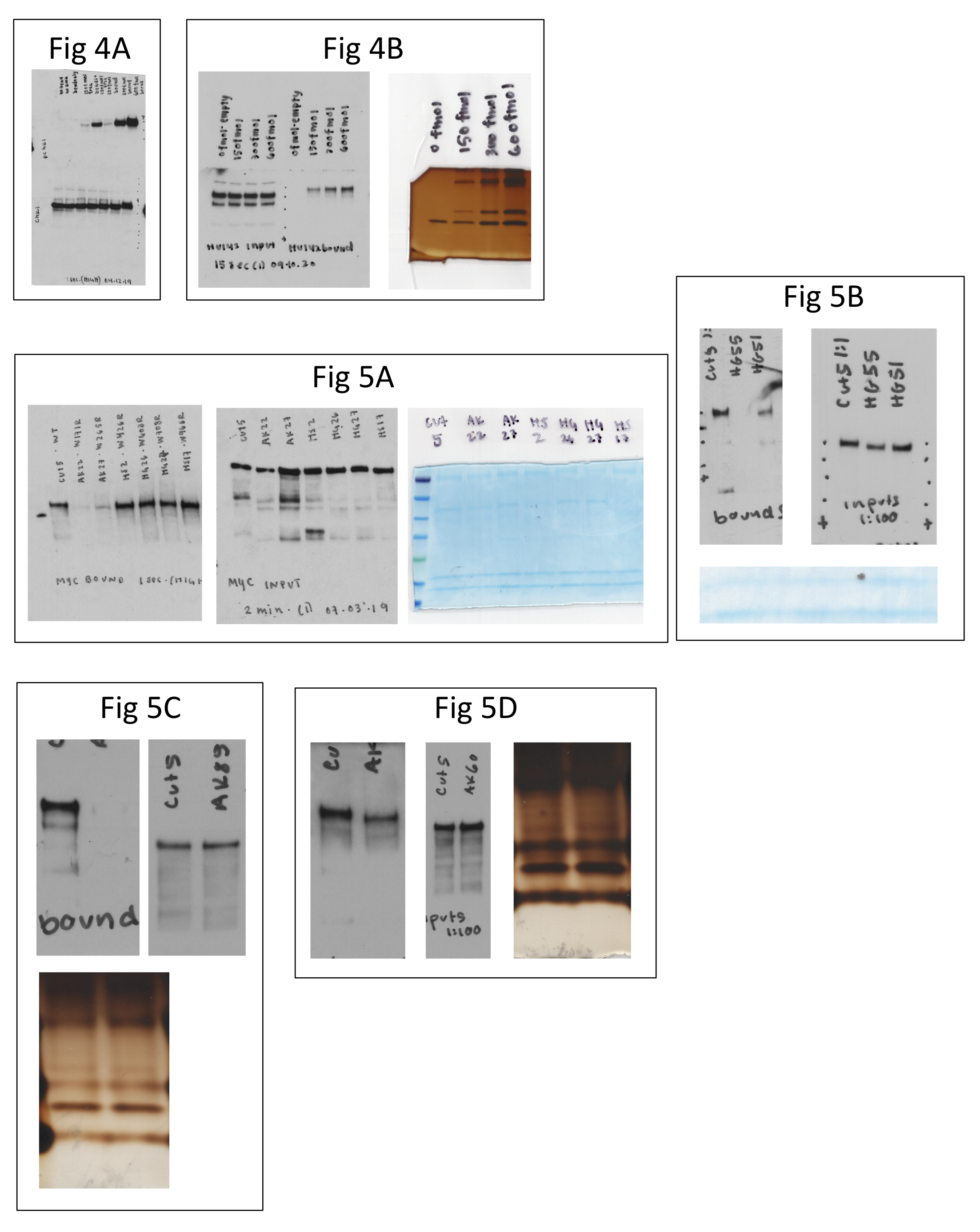
**

**Figure S2 continued.**

**
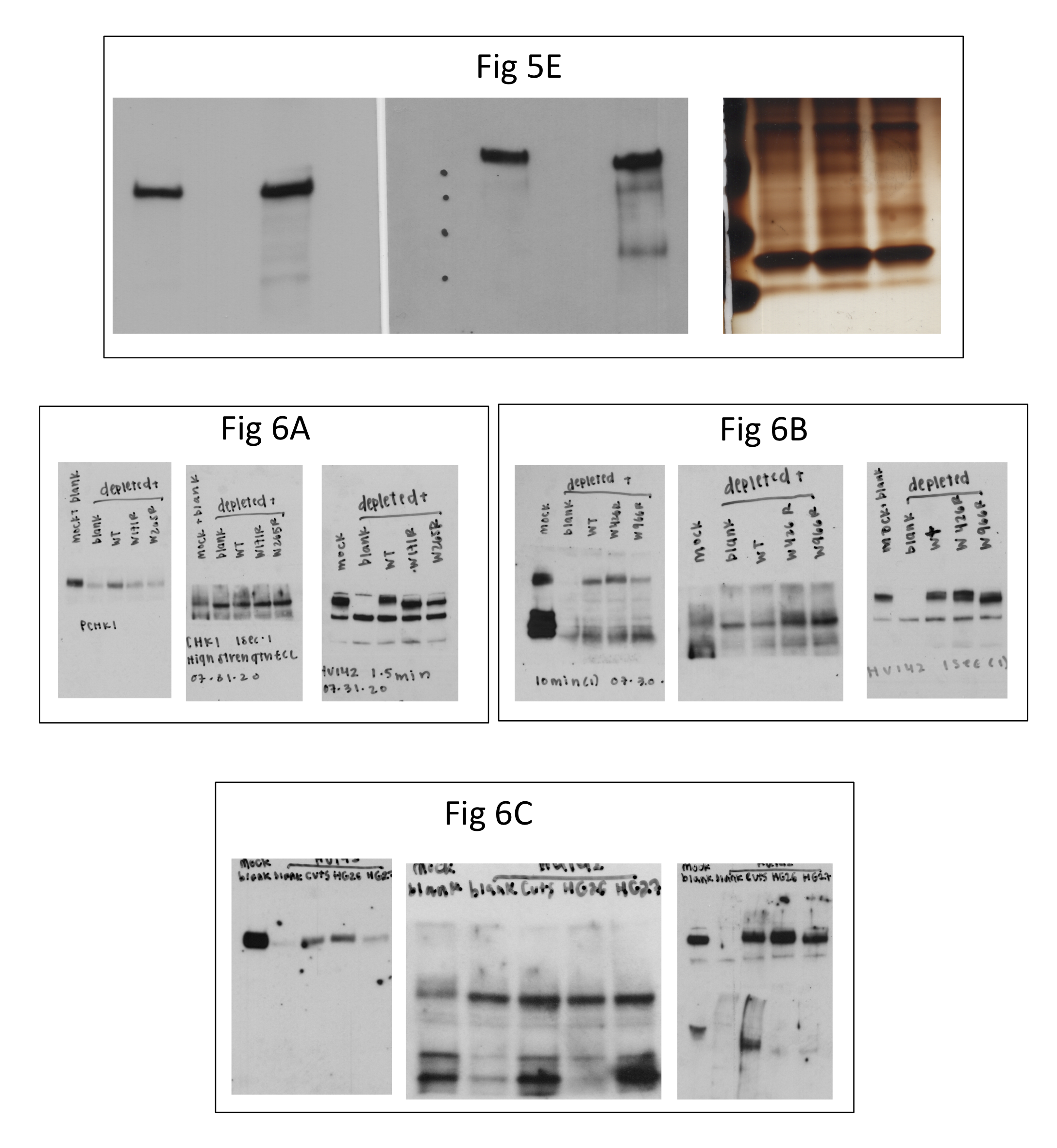
**

**Figure S2 continued.**

**
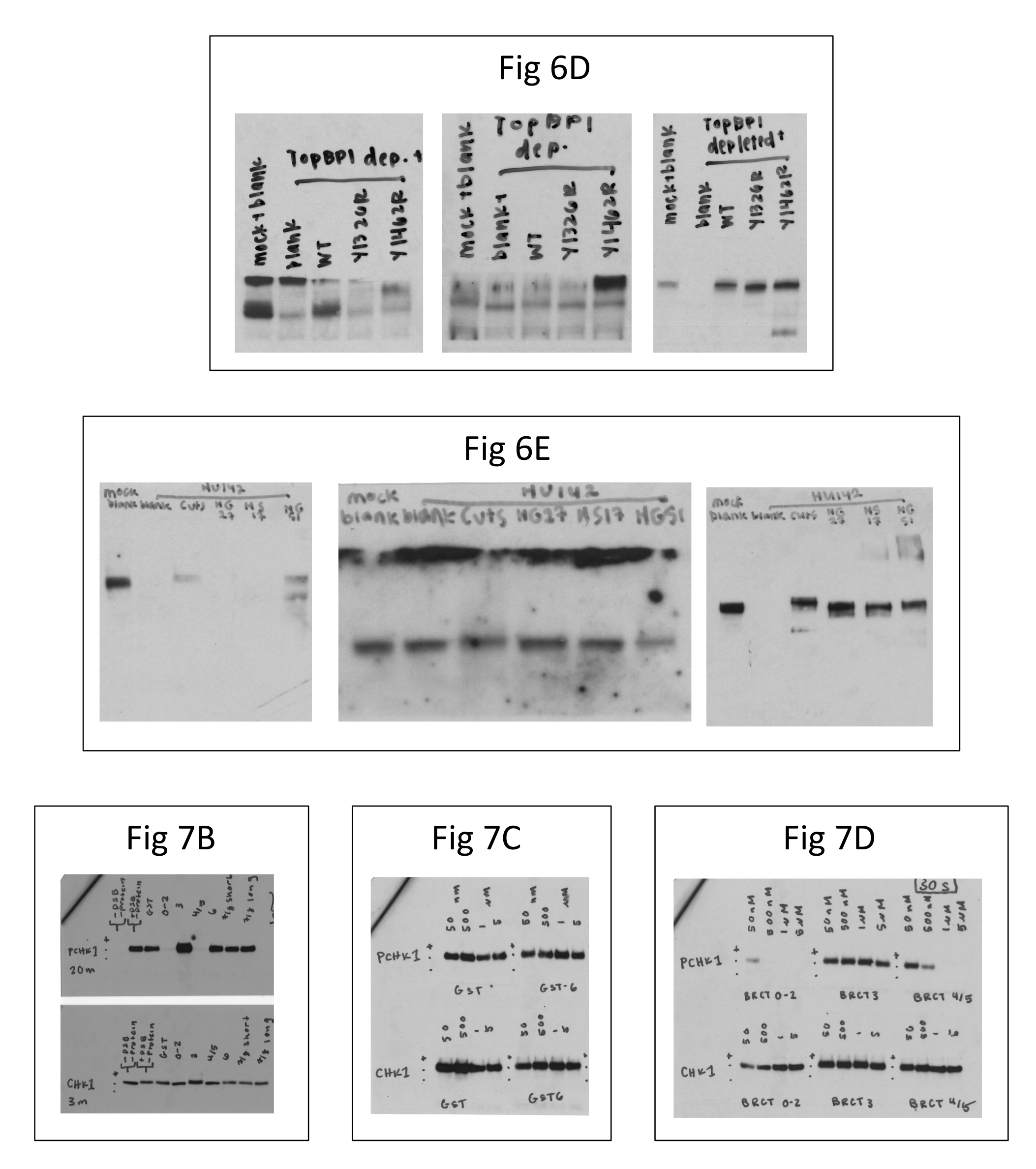
**

**Figure S2 continued.**

**
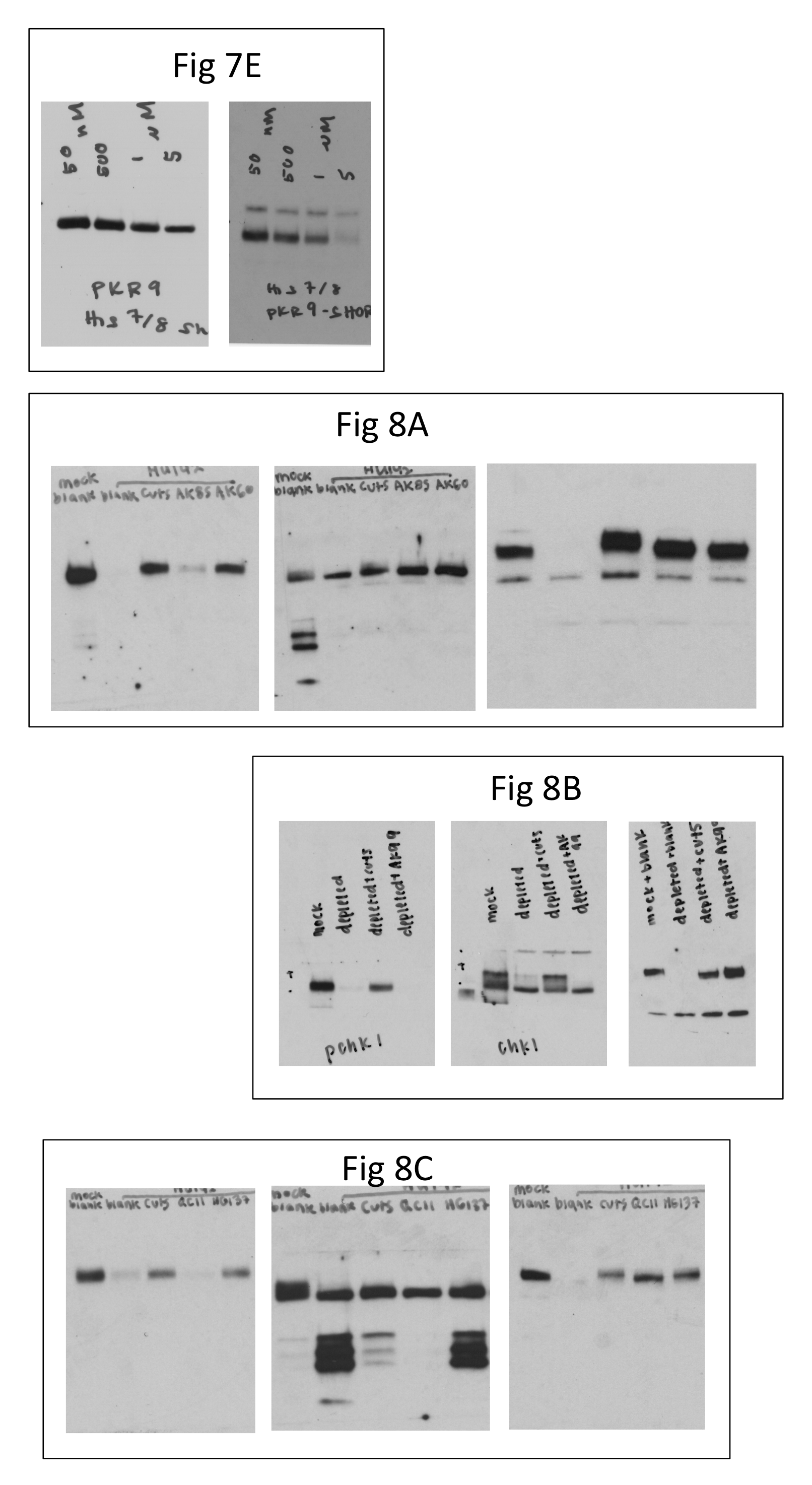
**

**Figure S2 continued.**

**
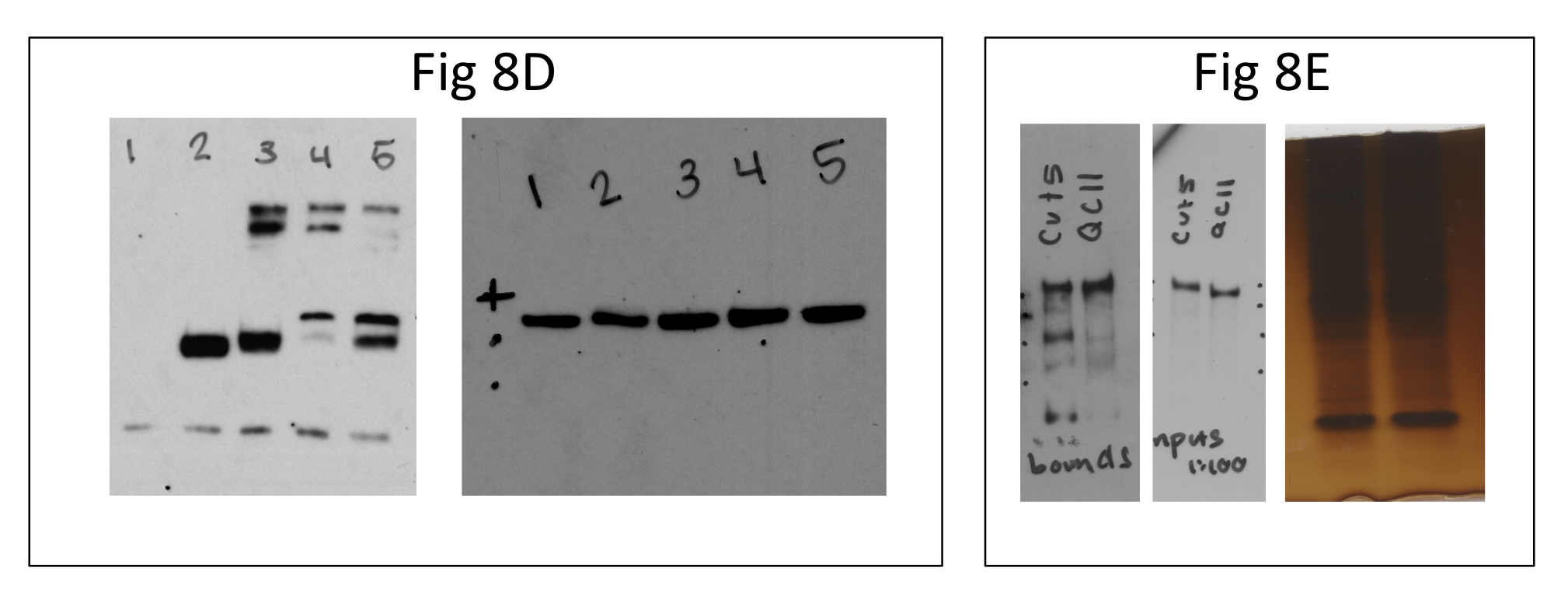
**

**Figure S2 contined.**
